# Concept of Planar Waveguide-Based *m* × *n* Terahertz Power Combiner

**DOI:** 10.3390/s26061965

**Published:** 2026-03-21

**Authors:** Rihab Hamad, Israa Mohammad, Thomas Haddad, Sumer Makhlouf, Tim Brüning, Andreas Stöhr

**Affiliations:** 1 Optoelectronics Department, University of Duisburg-Essen, 47057 Duisburg, Germany; 2 Microwave Photonics GmbH, 46047 Oberhausen, Germany

**Keywords:** terahertz, integrated optoelectronics, WR3-waveguide, 2D 2 × 2 WR3-power combiner, terahertz photodiode (THz-PD) arrays, slot bow-tie antenna

## Abstract

This paper presents the concept of a 2D *m* × *n* waveguide-based power combiner (PC) that is scalable with respect to the operating frequency band and number of input ports. To our knowledge, this work reports the first planar (2D) power combiner, where the input waveguide ports are distributed in two spatial dimensions to form an array, rather than arranged along a single linear (1D) axis as in conventional corporate or cascaded waveguide combiners. The novelty of the approach relies on using H-plane rectangular waveguide T-junctions and low-loss polarization twisters in between vertically stacked T-junctions to facilitate scalability. The work is motivated by the aim to coherently combine the output power of multiple modified uni-traveling carrier (MUTC) terahertz (THz) waveguide photodiodes (PDs) in a 2D array configuration. In the manuscript, the design of a 2 × 2 planar waveguide power combiner for the WR3 band (220–320 GHz) is reported, and it is also shown that this block can be further extended to *m* × *n* input ports. Full-wave numerical analysis of the proposed 2 × 2 power combiner shows a return loss of 11 dB at the output port and an average transmission coefficient of about −6.5 dB, i.e., an overall power combining efficiency of ~90%. Furthermore, to enable 2D photodiode array integration, the manuscript presents a new slot-bow tie antenna integrated MUTC photodiode for radiating the optically generated THz power from each PD vertically into the rectangular waveguide. The simulation results of reflection loss and insertion loss for the slot bow-tie antenna are shown to be better than 10 dB and 1.4 dB over the full WR3 band, respectively. To prove scalability of the power combiner concept w.r.t. the number of input ports, a 2 × 4 power combiner is also analyzed. Results reveal a return loss better than 10 dB from 225 to 318 GHz and a transmission coefficient of approximately −9.7 dB at 300 GHz, i.e., a power combining efficiency of ~85%.

## 1. Introduction

The terahertz (THz) spectrum, which ranges from 0.1 to 10 THz, is the space between microwaves and infrared regions, exhibiting distinct electromagnetic properties. Historically, the advancement of THz systems has encountered difficulties; however, recent years have witnessed significant advancements in sources, detectors, and system development [1,2]. Photonic-based sources provide a core technology for generating THz signals. These sources are commonly classified as THz lasers and laser-pumped photonic devices, including photomixers [3,4]. Photomixing, also known as optical heterodyne conversion, is a widely used technique for generating tunable continuous-wave (CW) THz radiation by illuminating an ultrafast photodiode or photoconductive device with two optical lasers whose frequency difference lies in the THz band [5]. Because the beat frequency between the lasers is converted into an electrical current oscillation in a photomixer, the output can provide high spectral purity and low phase noise qualities, especially valuable for high-resolution spectroscopy and coherent THz applications [6]. Current developments focus on enhancing output power, quantum efficiency, and integration with planar antennas or photonic platforms. The performance of chip-scale THz sources, including traditional photoconductive antennas (PCAs) and photodiodes (PDs), diminishes above 300 GHz due to transit time and RC-time limitations, hence restricting the saturation output power of small dies in the THz regime; for example, the maximum RF power obtained from a single THz PD is −3 dBm at 300 GHz due to its RC response time [7,8].

To achieve higher power levels of THz PDs, power combining techniques are commonly employed. THz power combiners can be typically classified according to the combining technique, such as: on-chip, free-space, and based-waveguide power combiners [9,10,11].

The on-chip power combiners integrate multiple THz-PDs on a single planar substrate using planar transmission lines, couplers, or corporate-feed networks, providing scalability and potential for integration. However, these on-chip combiners often suffer from high losses at high frequencies and limited bandwidth due to substrate modes, conductor losses, and impedance mismatches [9]. For instance, an on-chip T-junction was utilized to combine the output power of two THz PD chips at 300 GHz. Although on-chip combiners provide compactness and integration potential, this example underscores practical limitations: the enhancement in combined output power was noted only over a limited portion of the operating bandwidth, scalability to more ports or additional THz PDs is limited, and thermal constraints can further restrict performance. Additionally, the RC time constant of the devices limits high-frequency operation [12].

Free-space and waveguide-based combiners present alternative approaches for enhanced power combining, offering wider bandwidth and reduced loss. Free-space combiners, including beam splitters or quasi-optical grids, facilitate the spatial combination of THz PD outputs; nevertheless, they tend to be bulky and sensitive to precise alignment, hence constraining their use in compact integrated systems [10,11,13].

Power combiners based on waveguides in metallic structures can be categorized as E-plane, H-plane, and hybrid junctions. The implementation of these combiners becomes progressively more difficult as the number of input ports increases, owing to the demands of fabrication precision and the accumulation of insertion losses.

Choosing a suitable power combining method necessitates a balance between complexity, integration level, and frequency performance; for example, highly integrated on-chip power combiners, e.g., Wilkinson power combiner, may enhance compactness but are not quite scalable in terms of the number of input ports and also elevate loss and manufacturing challenges. Choosing a suitable power combining method necessitates a balance between complexity, integration level, and frequency performance; for example, highly integrated on-chip power combiners, e.g., Wilkinson power combiner, may enhance compactness but are not quite scalable in terms of the number of input ports and also elevate loss and manufacturing challenges. The motivation behind this work is to develop a power combiner for a 2D array of THz photodiodes. Because on-chip power combiners are lossy and do not provide wide operational bandwidths and free-space power combiners would become too bulky and would challenge optical coherency, a waveguide-based power combiner has been developed in this work. In general, waveguide-based power combiners can be differentiated into linear and planar arrangements. A linear waveguide power combiner (WR-PC) provides design simplicity and eases manufacturing by arranging the waveguide inputs along a single linear axis. However, as the number of input sources increases, the uneven distribution of the field leads to reduced combining efficiency and increased insertion losses [11]. For example, the linear eight-port rectangular waveguide power divider presented in [11], fabricated using low-cost CNC technology for heterodyne receiver array applications, exhibits a back-to-back insertion loss of 6.2 dB at 220 GHz and can only be used within a fraction of the full waveguide band. The significant insertion loss and bandwidth limitations illustrate the practical limitations of linear input arrangements in attaining effective power combining for multi-port THz power combiners over wide bandwidths. Although waveguide-type power combiner topologies are well established at microwave frequencies for planar array power combiners, their implementation using polarization twisters for developing a 2D planar array power combiner has not been studied well before. Furthermore, the direct implementation at terahertz (THz) frequencies is non-trivial. At THz frequencies, increased conductor and waveguide losses, stringent fabrication tolerances, and polarization control requirements significantly impact performance and bandwidth. In the manuscript, we not only propose a waveguide-type 2D planar array power combiner for THz frequencies but also show a possible integration path using photodiodes with on-chip THz antennas.

To address the limitations of linear input THz waveguide power combiners, including restricted scalability, uneven field distribution, reduced power combining and limited operational bandwidth, we propose a planar scalable 2 × 2 waveguide-based power combiner (WR-PC) (i.e., a combiner with four waveguide input ports and one waveguide output port) designed for operation over the full WR3-band (220–320 GHz). It is shown that, thanks to the incorporation of low-loss polarization twisters, the proposed architecture is scalable with respect to the number of input ports, allowing integration of larger 2D THz-PD arrays. To our knowledge, this work presents the first reported concept of a planar (2D) waveguide-based THz power combiner.

This paper is organized as follows. Section 2 delineates the design of a 2 × 1 waveguide power combiner, which constitutes the fundamental component for the proposed 2D 2 × 2 array configuration. Section 3 elaborates on this notion within a 2 × 2 configuration, addressing the comprehensive design and the integration of low-loss polarization twisters to guarantee mode and polarization alignment for effective power combining as well as for scalability. Section 4 addresses the integration of the power combiner with an array of THz photodiodes. This includes the design and analysis of new slot bow-tie antenna integrated waveguide-type MUTC photodiodes for vertical coupling of the optically generated THz signals into a waveguide input port of the power combiner. Finally, Section 5 summarizes the main findings and outlines potential directions for future work.

## 2. THz 2 × 1 WR3-Power Combiner

### 2.1. Design Principle

**Figure 1:**
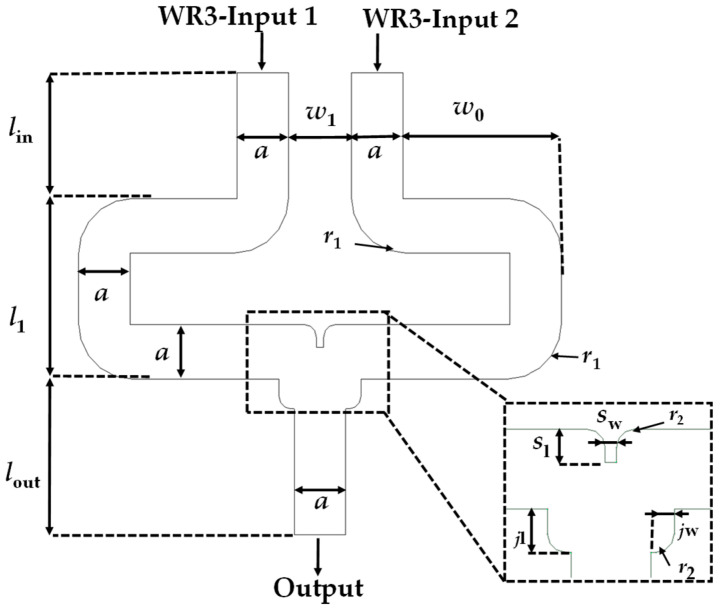
Proposed layout of 2 × 1 WR3-PC.

The 2 × 1 WR-PC is designed to merge the outputs of two THz photodiodes into a single waveguide output while minimizing reflection and insertion loss. The layout of the developed 2 × 1 WR3-PC is shown in Figure 1. The WR3-PC features two symmetrical standard WR3-waveguide inputs and one WR3-waveguide output to provide an in-phase interference of the input signals at the output section. This geometry follows the fundamental properties of a lossless passive three-port network T-junction [14].

The rectangular waveguide in this configuration has the standard cross-sectional dimensions of a WR3 waveguide, a × b = 864 × 432 µm^2^, for supporting only the propagation of the dominant TE_10_ mode. The junction area, in which the two input signals are combined, is optimized to ensure efficient combining, and the corners of the input waveguides are rounded to minimize the reflection of the EM-waves caused by the waveguide’s 90° sharp corners. Moreover, a septum element (denoted as *S*_w_ and *S*_l_) was adopted [15] within the combining region to reduce the undesired coupling between the input ports and to enhance the power combining efficiency. It is worth noting that a rectangular septum’s sharp corners may produce localized electromagnetic field concentrations, which increase the insertion losses (IL) and reflections. The septum’s fileted edges are intended to mitigate these effects and improve impedance matching.

To achieve impedance matching between input/output and the (*w*_1_), thereby minimizing signal reflection, the central waveguide section of width (*w*_1_, *w*_0_) is carefully optimized. On the other hand, (*l*_1_) refer to the path length where its lead the EM signals from the inputs to be merged at the combining region core, which tunes the phases of the input signals to provide a constructive interference at the output and has a value of about *λ*g.

To clarify the power-combining mechanism, the behavior of the proposed WR3-PC is analyzed using S-parameter power-wave theory. Let $a_{i}\;$ and $b_{i}\;$ denote the incident and outgoing waves at port i, respectively. For the three-port combining junction, the outgoing wave at the combining port (port 1) can be expressed as

(1) $$b_{1}\;=S_{12}a_{2}+S_{13}a_{3}$$

Assuming coherent excitation of equal amplitude and phase ($a_{2}=a_{3}=a_{0}\;$ the response of the junction is governed by its symmetry. Under this excitation condition, the reflected waves at ports 2 and 3 cancel due to destructive interference, while the transmitted waves toward port 1 add constructively. For an ideal symmetric junction with equal transmission coefficients $|S_{12}|$ = $|S_{13}|$ = 1/$\sqrt{2}\;$(−3 dB in amplitude), the output wave amplitude becomes

(2) $$b_{1}=a_{0}(\frac{1}{\sqrt{2}\;}+\frac{1}{\sqrt{2}})=\sqrt{2}a_{0}$$

The corresponding output power, proportional to ${|b_{1}|}^{2}$, is

(3) $$P_{out}\propto {|b_{1}|}^{2}=2{|a_{0}|}^{2}$$

Each input port contributes an incident power of ${|a_{0}|}^{2}$, resulting in a total input power $P_{in}$ = 2${|a_{0}|}^{2}$. Hence, under even-mode excitation, the combined output power satisfies $P_{out}$ =$\;P_{in}$.

It should be emphasized that this power conservation results from excitation-dependent reflection cancelation and constructive interference, rather than from the three-port junction being inherently lossless or perfectly matched. For odd-mode or unequal excitations, reflections are no longer canceled, and power combining efficiency degrades accordingly.

The waveguide section of width (*w*_1_, *w*_0_) is carefully optimized. On the other hand, (*l*_1_) refers to the path length, where it leads the EM signals from the inputs to be merged at the combining region core, which tunes the phases of the input signals to provide a constructive interference at the output and has a value of about *λ*g.

This design facilitates effective combination of the two-input power while preserving symmetry and phase integrity, which are paramount for high-frequency systems. The optimized design parameters for full-band and minimum IL are listed in Table 1, and the S-parameters will be discussed in the following section.

### 2.2. Numerical Analysis of THz 2 × 1 WR3-Power Combiner

**Figure 2:**
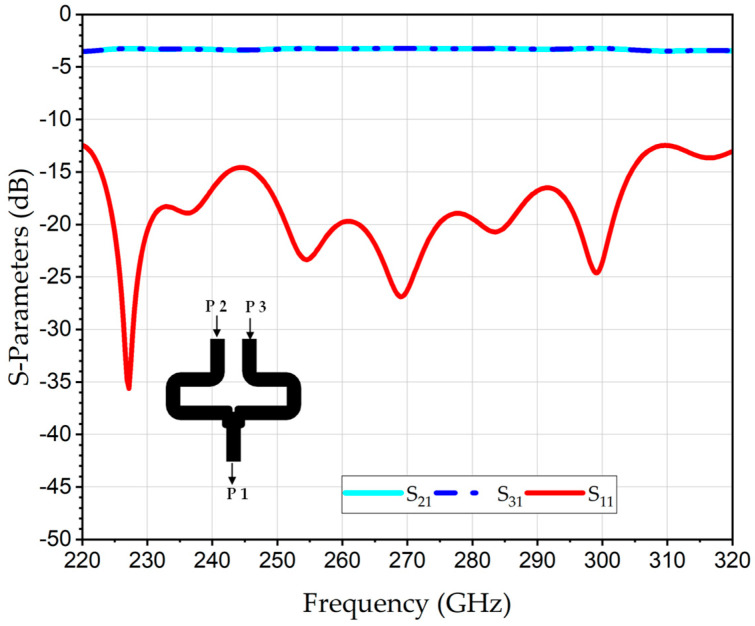
Numerically analyzed S-parameters of THz 2 × 1 WR3-PC.

The S-parameters of the 2 × 1 WR-PC across the complete WR3-band (220–320 GHz) are numerically evaluated using ANSYS ELECTRONICS DESKTOP (AED), version 2023, which is a finite-element method (FEM)-based 3D simulation platform for solving EM fields. Figure 2 represents the simulation result of the return losses (RL) at the output port (S_11_), which is better than 12 dB for the entire WR3-band; this is indicative of excellent impedance matching and minimal reflections. Likewise, the transmission coefficients from each input port to the output port, S_21_ and S_31_, revealed identical behavior with an average value of −3.2 dB. Since an ideal lossless 2 × 1 power combiner inherently exhibits a −3 dB splitting characteristic, the corresponding excess loss is approximately 0.2 dB.

Furthermore, while this approach could theoretically be extended to more ports, previous linear arrays of THz photodiodes (PDs) tend to be bulky, leading to higher fabrication and integration costs. Our compact 2 × 1 design thus provides a practical balance between performance and system complexity.

## 3. THz 2 × 2 WR3 Waveguide Power Combiner

The configuration of the proposed planar array 2 × 2 waveguide-based power combiner comprises two sections of power combining: the first section involves utilizing two parallel H-plane 2 × 1 WR3-PCs; thus, the formation of a 2 × 2 planar array of WR3 inputs is achieved in H-plane. The second section entails using a polarization twister to merge the in-phase outputs of the first section into an H-plane 2 × 1 WR3-PC. Two twisters are utilized to rotate the electric field by 90°.

### 3.1. Polarization Twister Design Principle

In order to build a 2 × 2 WR3-PC in a 2D planar input configuration, the electromagnetic performance at THz frequencies needs to be taken into consideration. A polarization twister is adopted to rotate the electric field orientation by 90°, enabling seamless mode transition between orthogonal waveguide sections (e.g., from H-plane to E-plane WR3 waveguides) [16,17].

The polarization rotation is achieved through a gradual geometrical transformation that adiabatically rotates the dominant TE_10_ mode electric field along the propagation direction. At the input, the WR3 waveguide supports a linearly polarized field aligned with one principal axis, which follows the rotation of the guiding boundaries as it propagates through the curved metallic structure formed by two symmetric circular arcs. All walls of the twister are metallic, ensuring well-defined boundary conditions and preserving modal confinement. The smooth curvature enforces a continuous rotation of the effective waveguide cross-section, resulting in a 90° rotation of the electric field at the output without exciting higher-order modes.

**Figure 3:**
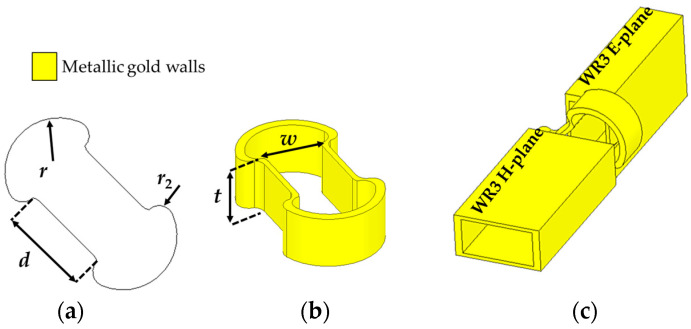
Structural schematic of the polarization twister designed for WR3 waveguide interfaces: (**a**) top view; (**b**) cross-sectional view of the junction; (**c**) 3D assembly view.

**Figure 4:**
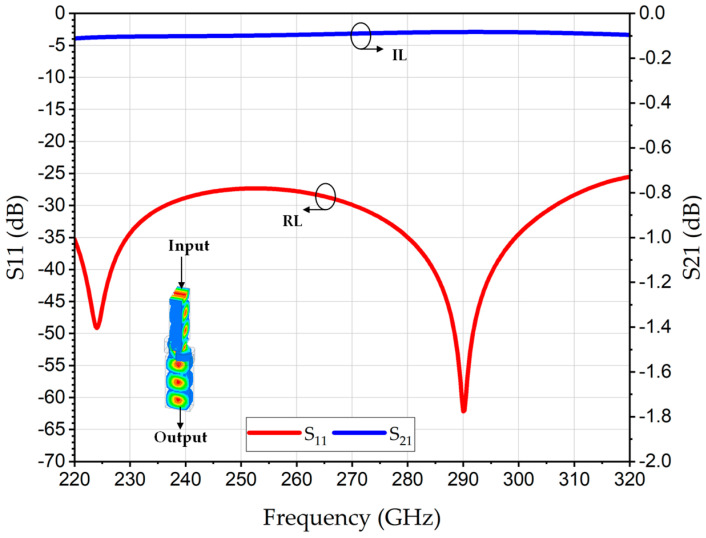
Simulated S-parameters of proposed polarization twister and the 90° polarization rotation of the E-field at frequency 275 GHz are dissipated in the inset.

Figure 3 represents the structural schematic of the proposed rectangular waveguide-based polarization twister designed for WR3-waveguide interfaces. The twister consists of two symmetrical circular metallic arcs forming the central propagation channel, with the top, bottom, and side walls metallic, and the curved regions providing the gradual rotation path. The key geometric parameters are: radius (*r*), the curvature of the edge, thickness (*t*), width (*w*), and a separation distance (*d*) defining the central channel through which most of the wave propagates. The radius (*r)* was optimized for smooth electrical field rotation between the WR’s E-plane and H-plane across the WR3-band. Moreover, the aperture’s parameters *d* and *w*, in addition to the twister’s thickness *t*, were optimized for low-loss transmission. The proposed polarization twister in this paper demonstrates an efficient, low-loss and full-band THz twister, overcoming the limited performance of the investigated THz twisters in the literature [16,17,18]. As shown in Figure 4, full-wave electromagnetic simulations confirm that the structure achieves an insertion loss (IL) (S_21_) of 0.2 dB and an S_11_ better than 25 dB over the entire WR3-band. The polarization illustrates the efficient 90° rotation of the electrical field at a frequency of 275 GHz and is given as an inset in Figure 4.

### 3.2. Planar Array 2 × 2 WR3-PC Design Principle

**Figure 5:**
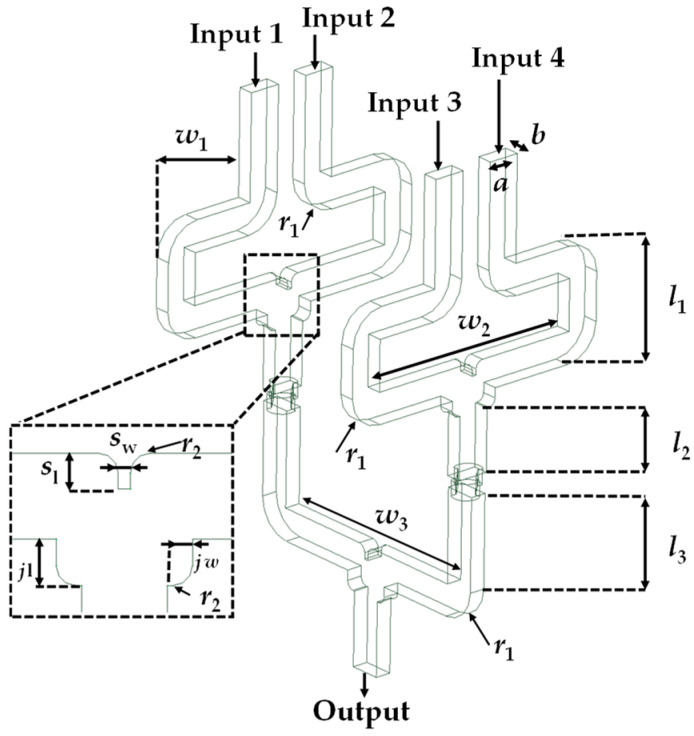
A 3D model of a 2 × 2 WR3-band WR3-PC employed in H-plane.

The configuration of the proposed planar array 2 × 2 WR3-PC is shown in Figure 5, which comprises four inputs arranged in parallel and one output. As mentioned before, the first section of the designed 2 × 2 PC involves using two 2 × 1 WR3-PC in parallel configuration; each WR3 output of these 2 × 1 is integrated with a polarization twister section for rotating the electric field by 90°, ensuring a seamless mode transition to an H-plane 2 × 1 WR3-PC. The power combiner’s overall length is about 14 mm; this 2D layout ensures in-phase power combining at the output, boosting the system’s overall efficiency, thereby achieving a wide operational bandwidth while maintaining minimal insertion loss.

Due to its topology-preserving and dimension-scalable design, the proposed power combiner is scalable w.r.t. the operating frequency band, as summarized in Table 2.

Furthermore, the same design concept can be extended to larger input arrays for sub-THz and terahertz power combining networks, as will be discussed in the following section. This renders it suitable for use in integrated high-frequency systems such as terahertz imaging, radar front ends, and free-space power combining.

**Figure 6:**
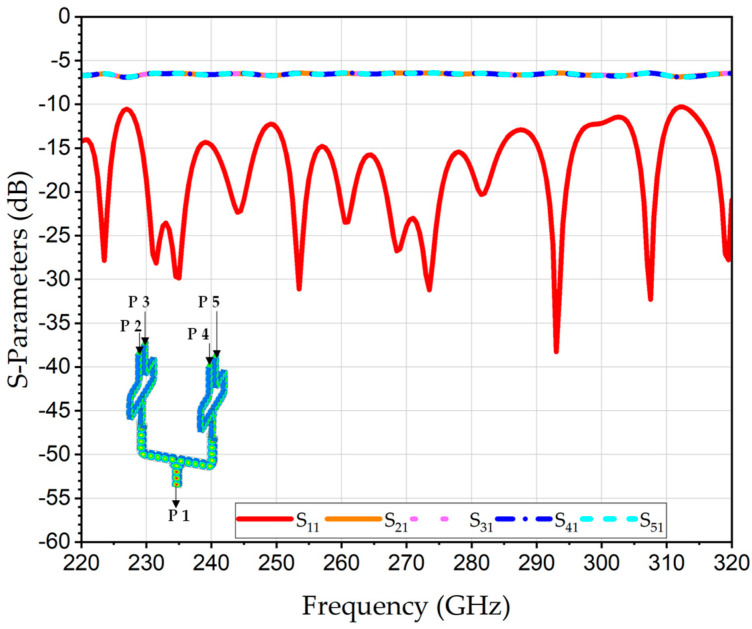
Simulated S-parameters of the 2D 2 × 2 WR3-PC. The distribution of the E-field between the inputs and the output at 275 GHz is given in the inset.

The proposed WR3-PC structure is designed to be compatible with high-precision CNC micro-machining using a split-block approach. This method facilitates the assembly of complex 3D structures like the polarization twisters. The design specifically incorporates rounded corners and fileted edges (e.g., *r*_1_ = 0.9 mm, *r*_2_ = 0.2 mm) to accommodate the finite radii of micro-milling bits. This ensures the simulated performance is representative of what can be achieved with physical tools. The simulations were conducted using high-conductivity metallic boundaries of gold. The simulated S-parameters of the 2D four-input WR3-PC are illustrated in Figure 6. The S_11_ is better than 10 dB over the entire WR3-band. The simulated average transmission coefficients of the S_21_, S_31_, S_41_ and S_51_ are about −6.5 dB. This value is aligned well with the theoretical combining loss of 6.02 dB of the four-port power combiner, thereby corroborating that the structure functions efficiently, exhibiting minimal excess loss of about 0.5 dB. In addition, the distribution of the E-field between the inputs and the output at 275 GHz is shown in the inset in Figure 6.

To provide a comprehensive overview of the potential of different approaches, Table 3 compares our numerical results with experimental results of several previously reported in-phase power dividers and combiners, considering key metrics such as frequency band, number of input ports, fractional bandwidth, insertion loss, efficiency, scalability, and integration with THz photodiodes. Compared with the designs reported in [11,15,19,20,21], the proposed combiner demonstrates a competitive fractional bandwidth (~37%), a low insertion loss (0.5 dB) as well as a high combining efficiency of ~90%. While some prior designs offer wider bandwidths or higher port counts, they either operate at lower frequencies or lack demonstrated scalability and photodiode integration. In contrast, the design proposed in this manuscript is conceptually scalable to larger 2D arrays of THz photodiodes with on-chip antennas, making it a promising candidate for high-power photonic terahertz systems.

### 3.3. Analysis of the Proposed 2D WR3-PC Structure for Multi-Input Scaling

**Figure 7:**
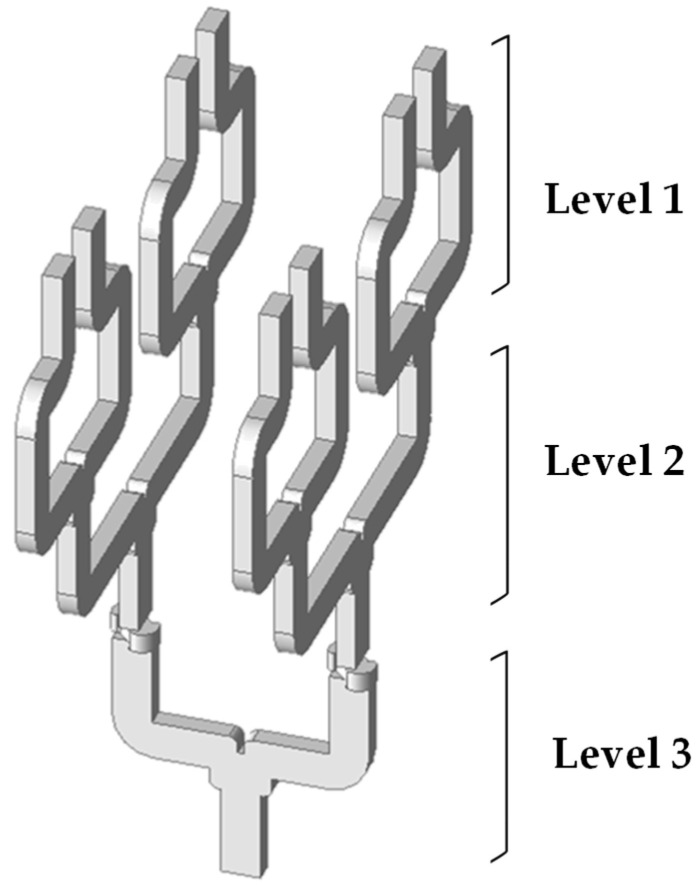
A 3D model of a 2 × 4 WR3 band WG-PC employing three levels.

To prove the scalability of the proposed WR-PC architecture in terms of input ports, a series of simulations and analyses were conducted on multiple configurations, including 2 × 1, 2D 2 × 2, and 2D 2 × 4 WR3-PC structures. In this work, a 2 × 2 waveguide-based power combiner has been developed, which can be extended to a *2* × *n* configuration by incorporating additional waveguide elements. As demonstrated in Figure 7, the extended structure with eight inputs exhibits defining a new level of combinations. Consequently, the structure includes three levels (levels 1 and 2 for power combining and level 3 for both E-field rotation and final combination to the output). As can be seen, levels 1 and 2 were developed in a linear configuration in order to reduce fabrication complexity. The implementation of the combiner through a split-block approach, utilizing a 1D configuration, markedly decreases the necessity for waveguide twists, thus facilitating alignment, reducing insertion losses, and enhancing manufacturability.

**Figure 8:**
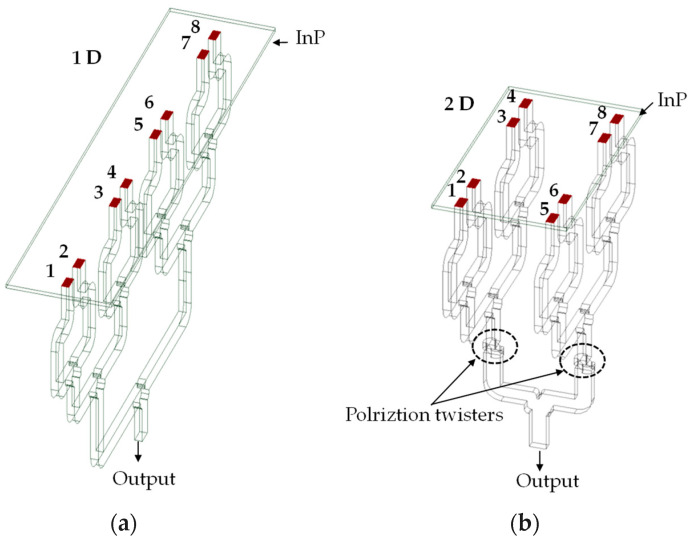
Schematic comparison between (**a**) a 1D (linear) power combiner and (**b**) the proposed 2D planar power combiner.

To our knowledge, this work reports the first planar (2D) power combiner, where the input waveguide ports are distributed in two spatial dimensions forming a planar array as can be seen from Figure 8. As mentioned above, the power combiner is designed for integration with an array of photodiodes. Since the wafer diameter in III/V technology is limited to typically 2–3 inches, one can integrate many more PDs using a planar array as compared to integrating the PDs along a linear array. As can be seen from Figure 8, our 4 × 1 power combiner section always operates in a single plane without the need for a polarization twister. However, for scalability, multiple 4 × 1 power combiners are combined. This combination can be done either in a linear fashion as shown in Figure 8a for an 8 × 1 linear array or in a planar fashion as shown in Figure 8b for a 4 × 2 power combiner. For the planar array power combiner consisting of multiple 4 × 1 sections, polarization twisters are needed as can be seen from Figure 8b.

To demonstrate the scalability of the proposed 2D WR3-PC architecture, Table 4 provides a framework for extending the design to 2 × n inputs. The total combining loss is estimated based on the simulated insertion loss per 2 × 1 combining stage of ~0.4 dB. It is noted that one can find slightly higher measured loss of ~0.6 for a similar 2 × 1 power combiner in the literature [19]. This value accounts for practical implementation effects that are not fully captured in ideal electromagnetic simulations, including finite metal conductivity, surface roughness, fabrication tolerances, assembly misalignment, and cumulative interface losses.

Table 4 estimates the cumulative combining loss as the number of combining levels increases, and evaluates the corresponding expected output power scaling for larger arrays. The reported output power values are expressed in a normalized form and are derived under the assumption of ideal coherent excitation at all input ports combined with a fixed per-stage loss. These values are intended to illustrate the scalability trend of the proposed architecture rather than to represent absolute output power, which ultimately depends on the available input power and implementation-specific losses.

This approach provides a conservative yet practical guideline for scaling 2D WR-PC structures for future high-power THz systems.

**Figure 9:**
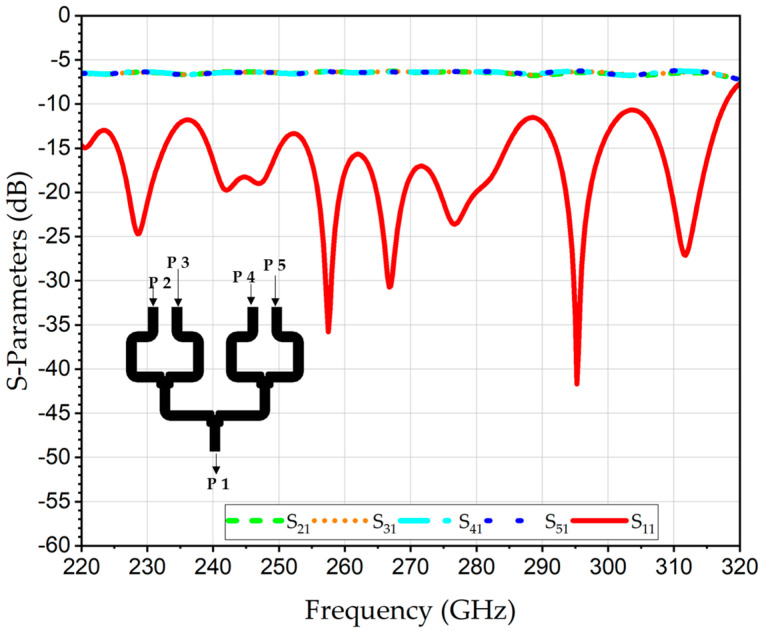
Simulated S-parameters of scalable 1D levels 1 and 2 of the 2 × 4 WR3-PC.

The levels 1 and 2 of the 2 × 4 WR3-PC were simulated, including the transmission coefficients from each input port to the output port S_21_, S_31_, S_41_, and S_51_, and RL at the output port S_11,_ are shown in Figure 9. The numerical analysis of the average transmission coefficients showed identical behavior with a value of −6.4 dB at 300 GHz.

**Figure 10:**
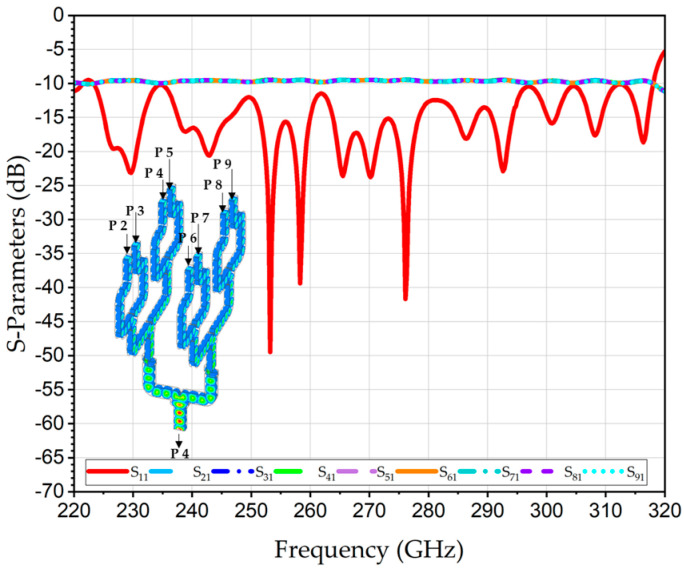
Simulated S-parameters of scalable 2 × 4 WR3-PC.

Furthermore, Figure 10 depicts the simulated S-parameters of the full 2 × 4 WR3-PC, including the transmission coefficients from each input port to the output port S_21_, S_31_, S_41_, S_51_, S_61_, S_71_, S_81_ and S_91_, as well as the RL at the output port S_11_. The average transmission coefficients are about −9.7 dB, and return loss at the output is about 10 dB covering the range 223–318 GHz.

## 4. Integration Concept with 2 × 2 THz PD Array

### 4.1. Integration Concept

**Figure 11:**
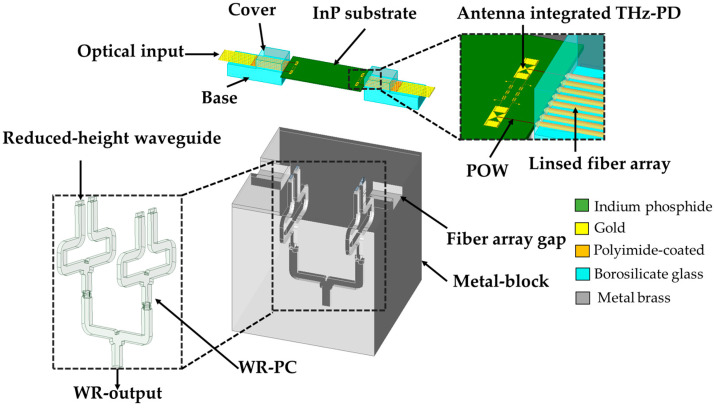
Proposed integration concept of a 2D THz 2 × 2 WR-PC block for WR3 frequency band. The colored inset in upper right corner shows the concept of the optical coupling with an InP-based PIC of monolithically integrated an array of 2 × 2 THz-PDs with antennas.

The proposed *m* × *n* WR-PC is capable of being integrated with a photonic-based THz input source. Figure 11 shows the concept for combining the output power of four on-chip THz PDs using a rectangular power combiner (WR-PC) with a 2 × 2 planar array of inputs in H-plane and one output.

A 2 × 2 array of THz PDs using a compact port inputs WR-PC, for a high-power photonic THz transmitter. In this concept, the THz PD array chip is mounted on the top of the 2 × 2 WR3-PC and precisely aligned with its WR3 inputs.

The photodiode array of four elements in this concept allows a direct optical-to-electrical conversion, minimizing the optical coupling losses and improving the responsivity [19,22]. An array of lensed optical fibers is used to simultaneously illuminate all the THz PDs, ensuring the generation of in-phase THz signals. These THz signals are then coupled into the inputs of the WRs-PC vertically by means of a slot bow-tie antenna. There is no need for a transition, neither at the input nor at the output. Finally, the THz signals will be efficiently guided through the WR-PC structure to be coherently combined at its WR-output. The antenna is developed to accomplish low insertion loss and consistent phase matching between the bow-tie slot antenna and the WR3-waveguide input port, while maintaining wideband operation.

**Figure 12:**
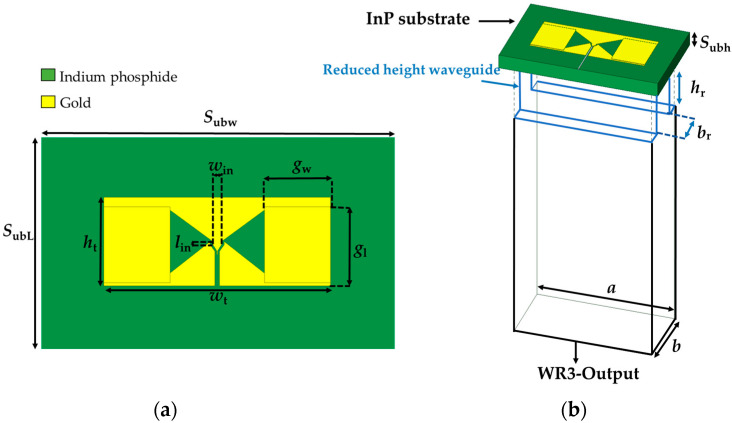
Proposed InP-based slot bow-tie-to-WR3 transition: (**a**) top view with design parameters; (**b**) integration concept to WR3-waveguide.

A slot bow-tie antenna is designed to radiate the THz signal vertically into the WR3 waveguide, as depicted in Figure 12a. The antenna is constructed on a 90 µm thick InP substrate, selected to optimize mechanical support, minimize dielectric loss at THz frequencies, and enhance coupling to the waveguide. In order to isolate the two electrodes of the bow-tie slot antenna and act as a short circuit at the designed frequency, the design configuration adopted the use of metal-insulator-metal capacitors (MIM) with a thickness of 2 µm. The numerical values of the design parameters are listed in Table 5.

The preliminary design simulated the slot bow-tie antenna over a WR3 waveguide utilizing HFSS. In these simulations, the input of the slot bow-tie antenna is referenced to an impedance of approximately 35 Ω, which corresponds to the effective radio-frequency output impedance of the MUTC photodiode–antenna interface. This value is used solely as a reference for evaluating the antenna-to-waveguide coupling efficiency in the full-wave simulations.

In practice, the antenna directly interfaces to the WR3 waveguide. It should be noted that a waveguide does not possess a lumped characteristic impedance in the same sense as a transmission line; instead, power transmission is governed by the propagation of the dominant TE_10_ mode. To improve matching between the low-impedance photodiode antenna and the waveguide, a reduced-height waveguide section is employed, which modifies the modal field distribution and effectively acts as an impedance-transforming transition. Under ideal simulation circumstances, the (S_21_) transmission from the input to the common WR3 port is about −3.22 dB at 300 GHz.

In the HFSS simulation, the input port was set to 35 Ω as a reference; the observed additional insertion loss arises mainly from structural losses in the antenna-waveguide system, including dielectric and conductor losses. As illustrated in Figure 12b, a reduced-height waveguide section is employed between the planar bow-tie slot antenna and the WR3 waveguide. Reducing the waveguide height decreases its characteristic impedance, bringing it closer to the antenna’s low input impedance, and thus acts as an effective quarter-wavelength impedance transformer. This design minimizes reflection and improves transition efficiency across the operational bandwidth [23,24].

### 4.2. Numerical Analysis of THz 2 × 2 THz PD Array

**Figure 13:**
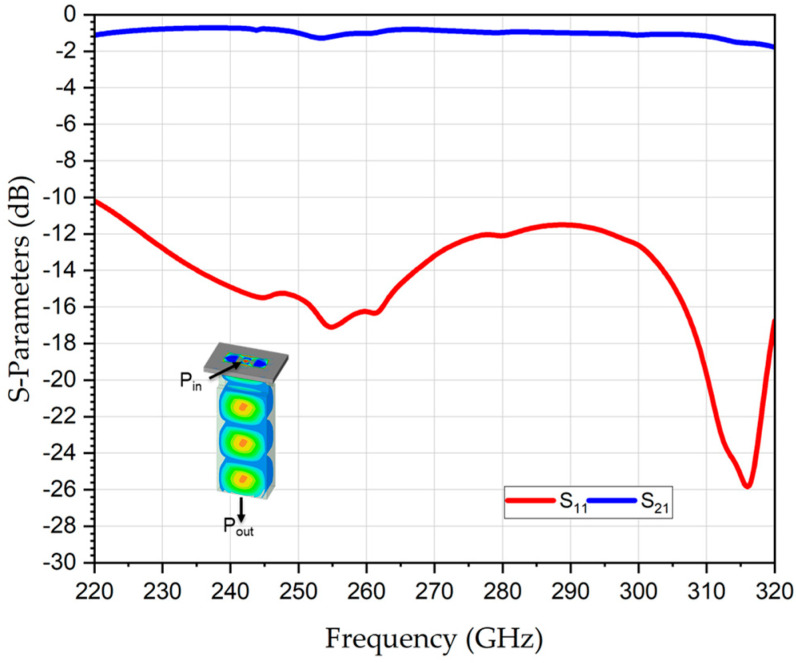
Simulated S-parameters of slot bow-tie antenna on WR3-waveguide aperture. The inset shows the E-field distribution from the antenna chip to the waveguide at 275 GHz.

The electrical performance of the InP-based slot bow-tie-to-WR3 waveguide was simulated via HFSS. The simulated S-parameters are presented in Figure 13. The IL is found to be 1.4 dB on the major part of the WR3-band. The corresponding RL (S_11_), at the input port, is better than 10 dB over the full band.

**Figure 14:**
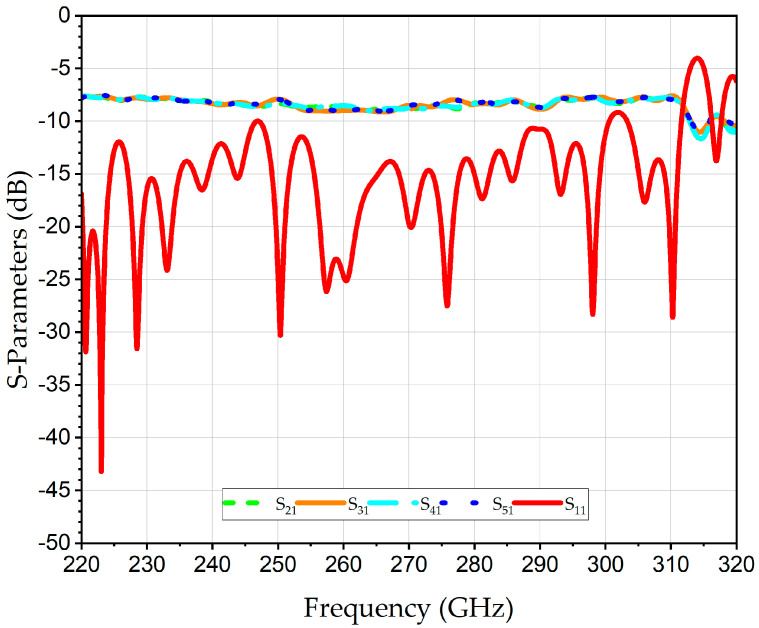
Simulated S-parameters of the integration of THz-PD array with planar array of 2 × 2 WR3-PC.

For the integration of the InP-based THz-PD array with the 2D four-port WR3-PC, Figure 14 reveals the simulated S-parameters from each slot bow-tie antenna transition to the output port S_21_, S_31_, S_41_ and S_51_, as well as RL at the output port. The simulated transmission coefficients show a value of approximately −7.6 dB at 300 GHz, which is higher than the transmission coefficients of a 2 × 2 WR3-PC because of the additional loss of the slot bow-tie-to-WR3 transition. Similar to the RL of the 2 × 2 WR3-PC, the RL of the integrated InP-based slot bow-tie array with the 2 × 2 WR3-PC also shows a similar performance better than 10 dB within the frequency range 220–300 GHz.

## 5. Conclusions

This paper presents the concept of a rectangular-waveguide-based power combiner for coherently integrating the output power from a 2D array of MUTC THz photodiodes. It is shown that the concept is scalable with respect to the number of input ports *m* × *n* thanks to using vertically stacked H-plane rectangular waveguide T-junctions interconnected by low-loss polarization twisters. The developed power combiner supports full-band operation and is also scalable in terms of operational frequency band. From full-wave numerical electromagnetic simulations the return loss at the output waveguide for a 2 × 2 WR3-band (220–320 GHz) power combiner is found to be 11 dB on average over the full band. The average transmission coefficient is about −6.5 dB, corresponding to an overall THz power combining efficiency of approximately 90%. To demonstrate scalability, a 2 × 4 WR3-band power combiner has also been studied. Here, the average return loss was determined to be better than −10 dB over the full band. The average transmission coefficient is −9.7 dB at 300 GHz, corresponding to a power combining efficiency of ~85%.

To enable seamless integration of 2D MUTC THz photodiode arrays, a novel slot bow-tie antenna that is monolithically integrated with a MUTC photodiode has also been developed. The antenna efficiently radiates the optically generated THz power vertically into the rectangular waveguide with a reflection loss better than 10 dB and an insertion loss below 1.4 dB over the full WR3-band, making it well suited for planar integration.

Overall, the proposed waveguide-type power combiner provides a compact and scalable solution for full-band and low-loss terahertz power combining. It is emphasized that the present work is purely theoretical and simulation-based. Thanks to the development of a planar slot bow-tie antenna that can be monolithically integrated with MUTC THz photodiodes, it is shown that the output power of individual photodiodes in a 2D array can be combined using the developed power combiner. Experimental fabrication and measurement of the proposed THz photodiode chips and power combiner are currently underway and are planned as part of future work, including manufacturability optimizations to support large-scale planar photonic-integrated THz systems. Such a photonic THz generator block would be a key element in various applications, e.g., to effectively extend the wireless range in point-to-point THz communication systems or in THz scanners. Future work will focus on experimental validation and manufacturability optimizations to facilitate large-scale planar photonic integrated THz systems.

## Figures and Tables

**Table 1:** Numerical values of the design parameters of 2 × 1 WR3-PC.

Design	Parameter	Value (mm)
2 × 1 WR3-PC	*l*_in_	2
	*l*_1_	2.862
	*l*_out_	2
	*w*_0_	2.66
	*w*_1_	1
	*S*_w_	0.124
	*S*_l_	0.364
	*j*_l_	0.472
	*j*_w_	0.255
	*r*_1_	0.9
	*r*_2_	0.2

**Table 2:** Numerical values of design parameters of the scalable 2 × 2 WR-PC w.r.t. the frequency band.

Component	Parameter	Value (mm)		
		WR4-Band	WR3-Band	WR2.2-Band
Twister	*r*	0.41	0.37	0.23
	*d*	0.69	0.618	0.38
	*w*	0.53	0.4717	0.29
	*t*	0.43	0.43	0.28
	*r*_0_	0.1	0.1	0.05
2 × 2 WR3-PC	*l*_1_	4	2	2.1
	*l*_2_	2.7	1.7	1.2
	*l*_3_	2	1.9	1.38
	*w*_1_	2.8	2.5	1.3
	*w*_2_	6.41	7.8	2.88
	*w*_3_	8.41	7.4	4.38
	*S*_w_	0.126	0.124	0.1
	*S*_l_	0.364	0.364	0.264
	*j*_l_	0.42	0.472	0.35
	*j*_w_	0.23	0.255	0.151
	*r*_1_	1.2	0.9	0.6
	*r*_2_	0.2	0.2	0.1

**Table 3:** Comparison of the proposed 2D WR-PC with previously reported in-phase dividers/combiners. Metrics include frequency band, number of input ports, fractional bandwidth, insertion loss, efficiency, size, scalability to 2D arrays, and THz photodiode integration.

Ref.	Frequency Band (GHz)	No. of Ports	BW (%)	IL (dB)	Efficiency (%)	Size	Scalability to 2D Arrays	Integration with THz-PDs
[20]	220–320	2	~36	0.16	—	~3.9 mm	Conceptually scalable	Designed for THz-PD arrays
[15]	220–330	2	41	1.2	—	~30 mm	—	—
[11]	Centered at ~220	8	~6.8	~6.2	—	—	—	—
[19]	93-107	4	~4.3	<1.2	~89	—	—	—
[21]	1–3	8	~20	—	—	424 × 49 × 1.56 mm^3^	—	—
This Work	220–320	4	~37	0.5	~90	14 mm	scalability theoretically validated	monolithically integrated

Note: Results for this work are numerical; all others are experimental. — not mentioned.

**Table 4:** Scalability of the 2D WR-PC architecture.

Nr. of Inputs (n)	Nr. of 2 × 1 PCs	Nr. of Polarizer	Nr. of Levels	Tot. Combining Loss (dB) = Nr. Levels × 0.4 dB	Relative Output Power Increase w.r.t. Single Input (dB)
2	1	0	1	0.4 dB	2.6
4	3	2	2	0.8 dB	5.2
8	7	2	3	1.2 dB	7.8
16	15	2	4	1.6 dB	10.4
32	31	2	5	2 dB	13
n	n − 1	2	$\log_{2}\left(n\right)$	$0.4\cdot \log_{2}\left(n\right)$	$\left({10\cdot \log}_{10}\left(n\right)\right)-0.4\cdot \log_{2}\left(n\right)$

The reported output power represents the relative power increase compared to a single input, excluding the absolute input power.

**Table 5:** Design parameters of the slot bow-tie-to-WR3 waveguide.

Parameter	Value (µm)
*h*_r_	380
*b*_r_	240
*S*_ubh_	90
*S*_ubw_	1300
*S*_ubL_	800
*w*_in_	20
*l*_in_	17
*g*_w_	240
*g*_l_	230
*h*_t_	268
*w*_t_	796

## Data Availability

The original contributions presented in this study are included in the article. Further inquiries can be directed to the corresponding authors.
